# Clinical Machine Learning Model for Predicting Pathological Complete Response in Patients with Esophageal and Gastroesophageal Junction Adenocarcinoma After Trimodality Therapy

**DOI:** 10.1245/s10434-026-19557-6

**Published:** 2026-04-19

**Authors:** Kohei Yamashita, Evan Kwiatkowski, Matheus Sewastjanow-Silva, Jane E. Rogers, Katsuhiro Yoshimura, Melissa Pool Pizzi, Qiong Gan, Jenny J. Li, Rebecca E. Waters, Mariela Blum Murphy, Masaaki Iwatsuki, Wayne L. Hofstetter, Aileen Chen, Ying Yuan, Jaffer A. Ajani

**Affiliations:** 1 Department of Gastrointestinal Medical Oncology, University of Texas M. D. Anderson Cancer Center, Houston, TX USA; 2 Department of Gastroenterological Surgery, Graduate School of Medical Sciences, Kumamoto University, Kumamoto, Japan; 3 Department of Biostatistics and Data Science, University of Texas Health Science Center at Houston, Houston, TX USA; 4 Department of Pharmacy Clinical Programs, University of Texas MD Anderson Cancer Center, Houston, TX USA; 5 Department of Pathology, University of Texas MD Anderson Cancer Center, Houston, TX USA; 6 Department of Thoracic and Cardiovascular Surgery, University of Texas MD Anderson Cancer Center, Houston, TX USA; 7 Department of Thoracic Radiation Oncology and Health Services Research, University of Texas MD Anderson Cancer Center, Houston, TX USA; 8 Department of Biostatistics, University of Texas MD Anderson Cancer Center, Houston, TX USA

## Abstract

**Background:**

Accurate prediction of pathological complete response (pCR) after preoperative chemoradiation therapy, followed by surgery (trimodality therapy) in esophageal adenocarcinoma (EAC) and gastroesophageal junction adenocarcinoma (GEJAC) may improve clinical decision-making and patient counseling before esophagectomy. This study aimed to develop predictive models for pCR after trimodality using machine learning (ML) approaches.

**Patients and Methods:**

A total of 569 patients with EAC and GEJAC who received trimodality therapy at MD Anderson Cancer Center between 2002 and 2022 were included. Clinicopathological characteristics and survival benefit of patients who achieved a pCR were reviewed via descriptive and survival analyses. Subsequently, ML models based on clinical variables were employed to predict pCR, including BART, random forest, and XGBoost, logistic regression, and LASSO.

**Results:**

pCR was achieved in 132 patients (23.2%). Poorly differentiated tumors, tumors with signet ring cell component, higher T stage, higher clinical stage, residual tumor on biopsy after chemoradiation, and higher SUVmax on positron emission tomography-contract tomography (PET-CT) after chemoradiation were significantly associated with non-pCR. pCR patients had significantly longer overall survival (OS) and relapse free survival (RFS) compared with non-pCR patients (median OS, 10.40 versus 4.42 years, log-rank *p* = 0.0041; median RFS, 10.40 versus 2.35 years, log-rank *p* < 0.0001). The random forest model showed the highest predictive ability for pCR with an AUC value of 0.702 among the employed models.

**Conclusions:**

This first exploratory study supports the validity and potential utility of ML-based models for predicting pCR after trimodality therapy in EAC and GEJAC. Further validation is warranted before clinical application.

Esophageal and gastroesophageal junction cancers represent a major global health burden, with the seventh highest cancer prevalence and sixth leading cause of cancer-related death in 2020.^1^ The incidence of esophageal cancer varies geographically between the two most common histologic subtypes, esophageal squamous cell carcinoma (ESCC) and esophageal adenocarcinoma (EAC).^2^ In the USA and Western Europe, ESCC is on the decline due to reduced tobacco and alcohol consumption. In contrast, EAC has been increasing in prevalence which is reportedly associated with obesity, gastroesophageal reflux disease, and Barrett’s esophagus.^3^ Thus, treatment of EAC and gastroesophageal junction adenocarcinoma (GEJAC) is of great interest in clinical practice.

Given the high malignant potential, multimodal therapy is employed to enhance the likelihood of curing localized disease. In the USA, trimodality therapy, which includes preoperative chemoradiation followed by radical surgery, is one of the standards to treat localized EAC and GEJAC. In previous clinical trials, approximately 23% of patients with EAC achieved a pathological complete response (pCR), defined as the absence of residual viable tumor cells in the surgically resected specimen (T0N0), after the trimodality therapy.^4^ pCR has been consistently associated with favorable long-term survival.^5,6^ The SANO trial demonstrated the noninferiority of active surveillance compared with immediate surgery among patients achieving a clinical complete response after preoperative chemoradiation.^7^ Similarly, accurate preoperative prediction of pCR may guide treatment and observation strategies and facilitate organ-sparing approaches. To date, several studies have indicated clinical factors related to pCR, such as tumor histological type,^8^ image findings,^9^ and endoscopic findings with biopsy results after chemoradiation.^10^ However, these studies had limitations in terms of accuracy, generalizability, and incorporation of multiple factors. Few studies discussed the clinical models that integrate these predictors.^11,12^ Further refinement and development of predictive models using emerging technologies are warranted to improve the accuracy and clinical applicability of such models.

Recently, various machine learning (ML) models have been developed for clinical diagnostic, treatment response, and prognostic use.^13–15^ ML can capture complex relationships, interactions, and nonlinear patterns in data compared with conventional logistic linear regression analysis. Thus, ML may provide better predictive performance given the complexity and heterogeneity of real-world data obtained from clinical practice.

In this study, we aimed to establish robust clinical parameter models to predict pCR after trimodality therapy in patients with EAC and GEJAC using various ML methods. We examined the clinicopathological factors related to pCR as well as the effects of pCR on patient survival. We then employed ML models and compared the predictive ability within the models to determine optimal prediction.

## Patients and Methods

### Study Design and Patient Selection

**Fig. 1:**
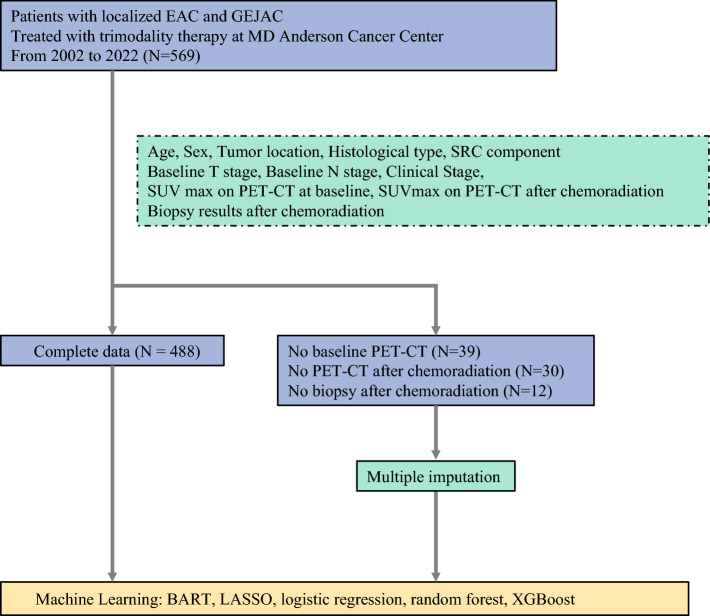
Patient selection and the number of imputed cases

This is a single-center retrospective observational cohort study at The University of Texas MD Anderson Cancer Center (MDACC). Patients were selected from the prospectively maintained clinical database which comprises information on patient demographics, clinicopathological features, treatment procedures, and survival. A total of 569 consecutive patients with localized EAC and GEJAC who were treated with trimodality therapy between June 2002 and February 2022 were identified in the database (Fig. 1). A total of 81 subjects were missing measurements: esophagogastroduodenoscopy (EGD) along with biopsy after chemoradiation for tumor restaging (*N* = 12), positron emission tomography and computed tomography (PET-CT) (*N* = 39), PET-CT after chemoradiation (*N* = 30). To avoid the assumptions regarding missing data necessary for a complete case analysis, which may introduce bias, and given that over 5% of the sample had a missing value potentially leading to a loss of power, we used multiple imputation by chained equations to analyze the full sample.^16^ This approach makes imputations on the basis of other observed covariate values for a given individual, accounts for the statistical uncertainty in the imputations, and has been used with analyses focusing on AUC as the summary statistic of interest.^17^ This study was conducted in accordance with the ethical principles outlined in the Declaration of Helsinki. The study protocol was approved by the institutional review board at MDACC (protocol number: PA12-1063), and all patients provided written informed consent for the subsequent use of their clinical data.

### Diagnosis, Treatment Course and Outcome Assessment

All the patients underwent clinical tumor staging via EGD, endoscopic ultrasonography (EUS), and baseline PET-CT prior to chemoradiation. Tumor location was identified on the basis of endoscopic findings. The Siewert classification was used to classify tumor location, with Siewert type I tumors considered as esophagus and type II tumors as GEJ.^18^ Baseline T stage, N stage, and clinical tumor stage were determined on the basis of EUS and PET-CT findings according to the 8th edition of the American Joint Committee on Cancer (AJCC) staging manual.^19^ The maximum standardized uptake value (SUVmax) of primary lesion on PET-CT was also recorded. Subsequently, patients received chemoradiation consisting of fluoropyrimidines plus platinum compounds or taxanes concomitantly with radiotherapy (RT). Total RT dose ranged from 45 to 50.4 Gy, delivered in 1.8 Gy daily fractions. Approximately 5–6 weeks after the completion of chemoradiation, an EGD along with biopsy was performed for tumor restaging, and the presence or absence of residual tumor was ascertained by pathological diagnosis. Additionally, PET-CT was performed to rule out distant metastases after chemoradiation and to measure the therapeutic effect, including the SUVmax of the primary tumor. Radical esophagogastrectomy with standard lymphadenectomy was ultimately performed, using surgical techniques such as Ivor-Lewis esophagectomy or total gastrectomy, depending on the location and extent of the tumor. Pathological assessment of the surgically resected specimen was conducted by institutional experienced pathologists. A pCR was defined as the absence of residual viable tumor cells in the surgically resected specimen (ypT0N0M0).

### Machine Learning Models

The ML models utilized the following clinical information: age, sex, tumor location, histological type, presence of signet ring cell (SRC) component, baseline TNM stage, biopsy results after preoperative CRT, SUVmax on PET-CT at baseline and after preoperative CRT. We established clinical models that predict pCR after trimodality therapy by performing ML methods. Decision-tree based machine learning methods, which have advantages in interpretability and computational efficacy over other machine learning techniques, are popular for classification modeling.^20^ Bayesian Additive Regression Trees (BART),^21^ random forest,^22^ and extreme gradient boosting (XGBoost)^23^ implement hierarchical decision rules. These can capture complex relationships between predictors and outcome and belong to the class of ensemble methods, which aggregate results from multiple models to improve accuracy and robustness. Random forest uses a bootstrap aggregation technique (i.e., bagging) which considers a random subset of predictors for each split in the tree-building process.^24^ XGBoost grows decision trees sequentially rather than using bootstrapping (i.e., boosting).^23^ Bayesian Additive Regression Trees (BART) uses a regularizing prior to prevent overfitting in a technique similar to boosting.^21^ Traditional statistical analyses are used as a comparison to these tree-based machine learning techniques. In addition to logistic regression,^25^ we considered Least Absolute Shrinkage and Selection Operator (LASSO),^26^ a variable selection technique built upon the regression framework which improves estimation by subsetting the predictor variables based on relevance. Each ML was performed according to the previous literatures and each method was trained and validated using 10-fold cross-validation, which ensures that the data used to evaluate the model is independent from the data used to train the model and is the most common model validation technique focused on the generalizability of the predictive model.^27^

### Statistical Analysis

Descriptive analyses were conducted to characterize the study population and provide an overview of variables related to pCR. Characteristics of pCR and non-pCR patients were compared using the *t*-test for continuous variables and the chi-square test for categorical variables. Univariate logistic regression analyses for predicting pCR were performed using variables of clinical interest. Overall survival (OS) was calculated as the period from the date of surgery to the date of event (death from any cause) or last follow-up date. Relapse-free survival (RFS) was calculated as the period from the date of surgery to the date of event (recurrence or death from any cause). Kaplan–Meier curves visualized the trends for OS and RFS, and the log-rank test was used to compare survival between pCR and non-pCR patients. All statistical tests were two-sided, and *p* < 0.05 was considered statistically significant. In addition to the ML algorithms described above, all statistical analyses were performed in the R statistical environment (v 4.3.0).

## Results

### Relationships Between Clinicopathological Factors and pCR

A total of 132 out of 569 patients achieved a pCR, corresponding to an overall pCR rate of 23.2%. The relationships between clinicopathological factors and pCR are summarized in Table 1. Among the patients who achieved pCR, 87.1% were male and 12.9% were female. The mean age of patients with pCR was 59.6 years (Standard deviation [SD] = 9.90), ranging from 26 to 77 years. There was no statistical difference between patients who achieved pCR and those who failed to achieve pCR (non-pCR) with respect to age and sex. Similarly, tumor location, baseline N stage, and baseline SUVmax on PET-CT were not statistically different between the two groups. On the other hand, poorly differentiated histological type (*p* = 0.0356), tumors with SRC components (*p* = 0.00212), higher baseline T stage (*p* = 0.00195), residual carcinoma on biopsy after chemoradiation (*p* < 0.001), and higher SUVmax on PET-CT after chemoradiation (*p* < 0.001) were significantly associated with non-pCR.

**Table 1:** Comparison of characteristics between pCR and non-pCR

Variables	pCR (*N*=132)	Non-pCR (*N*=437)	*P*-value
*Age*			
Mean (SD)	59.6 (9.90)	60.0 (9.81)	0.639
Median [Min, Max]	62.0 [26.0, 77.0]	61.0 [26.0, 80.0]	
*Sex*			
Male	115 (87.1%)	399 (91.3%)	0.209
Female	17 (12.9%)	38 (8.7%)	
*Tumor location*			
Esophagus	74 (56.1%)	247 (56.5%)	1
GEJ	58 (43.9%)	190 (43.5%)	
*Histological type*			
Well/moderately differentiated	73 (55.3%)	194 (44.4%)	0.0356
Poorly differentiated	59 (44.7%)	243 (55.6%)	
*Tumor with SRC component*			
Yes	10 (7.6%)	85 (19.5%)	0.00212
No	122 (92.4%)	352 (80.5%)	
*Baseline T stage*			
T1/T2	24 (18.2%)	36 (8.2%)	0.00195
T3/T4	108 (81.8%)	401 (91.8%)	
*Baseline N stage*			
N0	56 (42.4%)	189 (43.2%)	0.305
N1	65 (49.2%)	192 (43.9%)	
N2/N3	11 (8.3%)	56 (12.8%)	
*Clinical stage*			
I/II	19 (14.4%)	25 (5.7%)	0.00255
III	102 (77.3%)	355 (81.2%)	
IVA	11 (8.3%)	57 (13.0%)	
*Biopsy after chemoradiation*			
Negative for carcinoma	123 (93.2%)	325 (74.4%)	< 0.001
Positive for carcinoma	4 (3.0%)	96 (22.0%)	
Missing	5 (3.8%)	16 (3.7%)	
*Baseline SUVmax on PET CT*			
Mean (SD)	12.6 (9.88)	12.4 (8.06)	0.868
Median [Min, Max]	10.3 [0, 58.0]	10.7 [0, 48.5]	
Missing	10 (7.6%)	27 (6.2%)	
*Post CRT SUVmax on PET CT*			
Mean (SD)	3.72 (2.44)	4.78 (3.23)	< 0.001
Median [Min, Max]	4.10 [0, 9.30]	4.70 [0, 39.7]	
Missing	8 (6.1%)	32 (7.3%)	

*pCR* pathological complete response, *GEJ* gastroesophageal junction, *SRC* signet ring cell, *CRT* chemoradiation therapy, *SUV* standardized uptake value

### Survival Analysis

We next performed survival analysis to confirm the benefit of pCR on patient survival and disease recurrence. The median follow-up period for all eligible patients was 3.64 years. During this follow-up period, 50 pCR patients (37.9%) and 202 non-pCR patients (46.2%) have died. A total of 53 pCR patients (40.2%) and 249 non-pCR patients (57.0%) had RFS events (recurrence or death from any cause).

**Fig. 2:**
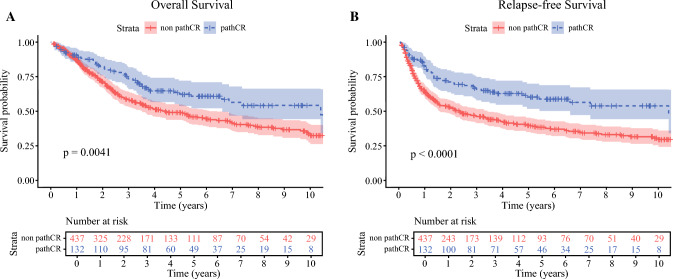
Impact of pathological complete response (pCR) on patient survival and recurrence after trimodality therapy. **A** Kaplan–Meier curves for overall survival in patients with pCR and non-pCR. **B** Kaplan–Meier curves for relapse free survival in patients with pCR and non-pCR

The Kaplan–Meier curves for OS and RFS are shown in Fig. 2. pCR patients demonstrated significantly longer OS compared with non-pCR patients (log-rank *p* = 0.0041), with the median OS of 10.40 years (95% confidence intervals [CI], 6.61–not estimable) and 4.42 years (95% CI, 3.52–6.40), respectively. Additionally, RFS of pCR patients was significantly extended compared with that of non-pCR patients (log-rank *p* < 0.0001), with median RFS of 10.40 years (95% CI, 6.61–not estimable) and 2.35 years (95% CI, 1.55–3.42), respectively.

### Univariate Logistic Regression Analysis for Predicting pCR

We next performed a univariate logistic regression analysis to examine the factors associated with pCR (Table 2). The logistic regression model for concerned variables demonstrated tumors with SRC component (Odds ratio [OR] = 0.34, 95% CI 0.16–0.65, *p* = 0.002) and higher SUVmax on PET-CT after chemoradiation (for a one-point increase in SUV max: OR = 0.86, 95% CI 0.80–0.94, *p* < 0.001) were significantly associated with non-pCR, while well-/moderately differentiated histological type (OR = 1.55, 95% CI 1.05–2.30, *p* = 0.028), T1/T2 stage (OR = 2.48, 95% CI 1.40–4.31, *p* = 0.001), clinical stage I/II (OR = 3.94, 95% CI 1.66–9.75, *p* = 0.002), no carcinoma detected on biopsy after chemoradiation (OR = 9.08, 95% CI 3.70–30.1, *p* < 0.001) were significantly associated with pCR.

**Table 2:** Univariate logistic regression analysis for predicting pCR

Variables	OR	95% CI	*P*-value
Age	1	0.98, 1.02	0.64
*Sex*			
Male	0.64	0.36, 1.21	0.16
Female	–	–	
*Tumor location*			
Esophagus	0.98	0.66, 1.46	0.93
GEJ	–	–	
*Histological type*			
Well-/moderately differentiated	1.55	1.05, 2.30	0.028
Poorly differentiated	–	–	
*Tumor with SRC component*			
Yes	0.34	0.16, 0.65	0.002
No	–	–	
*Baseline T stage*			
T1/T2	2.48	1.40, 4.31	0.001
T3/T4	–	–	
*Baseline N stage*			
N0	1.51	0.76, 3.21	0.26
N1	1.72	0.88, 3.65	0.13
N2/N3	–	–	
*Clinical stage*			
I/II	3.94	1.66, 9.75	0.002
III	1.49	0.78, 3.09	0.25
IVA	–	–	
*Biopsy after chemoradiation*			
Negative for carcinoma	9.08	3.70, 30.1	< 0.001
Positive for carcinoma			
Baseline SUVmax on PET CT	1	0.98, 1.03	0.85
Post CRT SUVmax on PET CT	0.86	0.80, 0.94	< 0.001

*OR* odds ratio, *CI* confidence interval, *pCR* pathological complete response, *GEJ* gastroesophageal junction, *SRC* signet ring cell, *CRT* chemoradiation therapy, *SUV* standardized uptake value

### Machine Learning Models for Predicting pCR

**Fig. 3:**
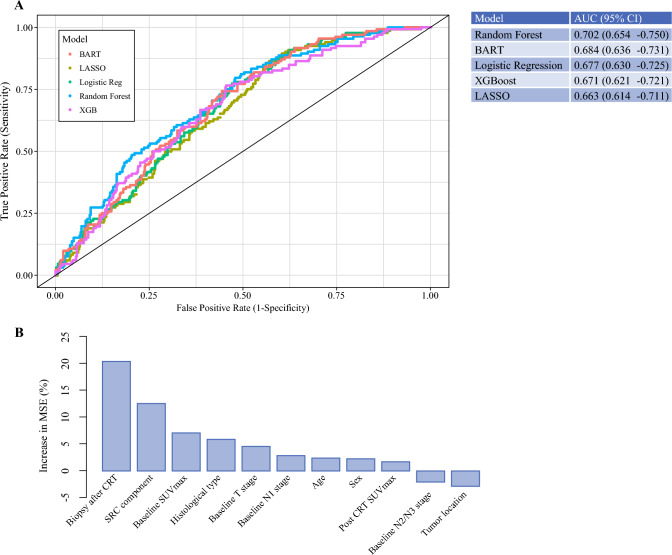
Predictive ability of the machine learning (ML) models. **A** Receiver operating characteristic (ROC) curves and area under the curve (AUC) values for the ML models. **B** Variable importance plot for the random forest model

Finally, we employed various ML models with the above variables to predict pCR. The receiver operating characteristic (ROC) curves of different ML models were described in Fig. 3A. All MLs exhibited similar ROC curves. The Area under the ROC curve (AUC) values of BART, LASSO, logistic regression, random forest and XGBoost were 0.684, 0.663, 0.677, 0.702, and 0.671, respectively. Random forest exhibited the largest AUC value among the models with AUC 0.702 (95% CI 0.654–0.750). Figure 3B demonstrates importance of the covariates from the random forest model as measured by increase in mean squared error (MSE) of predictions in the hypothetical situation where the values of that variable were randomly permuted.^28^ For example, if the values of Biopsy after CRT were permuted, the MSE of the predictions would increase by 20.4%. The five most important variables were Biopsy after CRT, SRC component, baseline SUVmax, histological type, and baseline T stage, which with the exception of baseline SUVmax were covariates also highly associated with pCR from the univariate associations of Table 2.

## Discussion

The present study aimed to establish a robust clinical ML model to predict pCR after trimodality therapy in patients with EAC and GEJAC. Our results demonstrated that the overall pCR rate was 23.2%. Patients who achieved a pCR had significantly longer OS and RFS, which are consistent with the previous literature. Notably, we employed various ML models using real-world data and showed potential predictive ability for pCR. This first exploratory research to utilize ML for the prediction of pCR after trimodality therapy will suggest an important implication to the field.

The efficacy of trimodality therapy in patients with localized EAC and GEJAC has been extensively documented in previous landmark clinical trials.^4^ Furthermore, several clinical trials consistently demonstrated a higher pCR rate with preoperative CRT compared with preoperative chemotherapy alone.^29^ Consequently, the trimodality therapy has been one of the preferrable options for patients with localized EAC and GEJAC globally.^30,31^ However, recent data from the ESOPEC trial demonstrated improved long-term survival with perioperative chemotherapy using the FLOT regimen compared with preoperative chemoradiation followed by surgery, raising questions regarding the optimal multimodal approach.^32^ Despite these findings, in real-world clinical practice, trimodality therapy continues to play an important role in the management of locally advanced disease, particularly for achieving durable local tumor control in selected patients. In this context, accurate prediction of pCR remains clinically meaningful, as it may help identify patients most likely to benefit from trimodality therapy and inform individualized treatment and surveillance strategies. Our real-world data further support the prognostic significance of pCR in terms of survival and recurrence, underscoring the potential value of predictive models in contemporary clinical decision-making. Further investigations are warranted to elucidate the specific factors influencing recurrence and prognosis in patients who have achieved pCR.

Given the current landscape, accurate prediction of pCR relies heavily on the quality and size of the training data used to develop predictive models. Among previous studies focusing on EAC and GEJAC,^33–35^ our study stands out with one of the largest real-world cohorts to date, strengthening the credibility of our findings. Our results substantiate earlier reports by demonstrating that tumors with SRC component and deeper tumor depth (T3/T4) are less likely to achieve pCR.^8,36^ Additionally, we demonstrated that negative biopsy results and lower SUVmax on PET-CT after chemoradiation were associated with a higher likelihood of pCR. Although a few studies indicate the limitations of pCR prediction with these modalities alone,^10,37^ our findings suggest that incorporating restaging procedures to assess treatment response following preoperative CRT in clinical practice is effective for predicting pCR. This study serves as an exploratory investigation, leaving room for further inquiry into additional predictors that may contribute to enhancing the predictive performance of the model.

Recently, there has been a growing exploration of ML for clinical applications in cancer treatment.^38–40^ In esophageal cancer, ML models have shown proficiency of diagnosis ability in tasks of image pattern recognition for various medical imaging modalities, such as endoscopic, radiological, and pathological images.^41–43^ Additionally, there are reports of predictive models that encompass multiple factors, such as predicting postoperative complications, recurrence, survival, and treatment efficacy.^44–48^ ML is considered promising owing to its ability to handle the heterogeneity and nonlinearity of real-world data. In our study, we employed five different ML models and compared their predictive ability. The tree-based machine learning methods performed similarly to logistic regression, which has been shown to occur when using a smaller number of strong predictor variables.^49^ Random forest had the highest AUC overall, demonstrating the potential of ML to address clinical prediction of pCR after trimodality therapy. Further exploration is needed to elucidate the factors affecting the predictive performance of different ML models and their applicability in clinical decision-making. Although not yet suitable for direct clinical implementation, the present study contributes to the identification of clinically relevant patient subgroups, hypothesis generation, and the advancement of biomarker and predictive model development in precision oncology.

We recognize several limitations in this study. First, this study is based on retrospective data from a single institution, which may introduce inherent biases and limit the generalizability of the findings. This limitation could be addressed by establishing prospective, multi-institutional cohorts. Second, although ML models provided insights in predicting pCR, there are limitations in establishing robust predictive models based on clinical data alone. Improvements in predictive accuracy may be achieved through incorporation of tumor molecular and imaging data to better capture biological heterogeneity, and integration of longitudinal treatment-response information beyond baseline characteristics. Such advances will be important to support the development of clinically applicable predictive models.

In conclusion, this study highlights the potential of ML models to predict pCR in patients with EAC and GEJAC who received trimodality therapy. The development of accurate predictive models of pCR in this context suggests important implications for treatment decision making, maintaining patient quality of life through organ preservation, and potentially improving patient outcomes.
